# The effects of N-terminal insertion into VSV-G of an scFv peptide

**DOI:** 10.1186/1743-422X-3-69

**Published:** 2006-09-02

**Authors:** Hanna Dreja, Marc Piechaczyk

**Affiliations:** 1 Institut de Génétique Moléculaire de Montpellier, UMR 5535, IFR122, CNRS, France

## Abstract

Recombinant retroviruses, including lentiviruses, are the most widely used vectors for both *in vitro *and *in vivo *stable gene transfer. However, the inability to selectively deliver transgenes into cells of interest limits the use of this technology. Due to its wide tropism, stability and ability to pseudotype a range of viral vectors, vesicular stomatitis virus G protein (VSV-G) is the most commonly used pseudotyping protein. Here, we attempted to engineer this protein for targeting purposes. Chimaeric VSV-G proteins were constructed by linking a cell-directing single-chain antibody (scFv) to its N-terminal. We show that the chimaeric VSV-G molecules can integrate into retroviral and lentiviral particles. HIV-1 particles pseudotyped with VSV-G linked to an scFv against human Major Histocompatibility Complex class I (MHC-I) bind strongly and specifically to human cells. Also, this novel molecule preferentially drives lentiviral transduction of human cells, although the titre is considerably lower that viruses pseudotyped with VSV-G. This is likely due to the inefficient fusion activity of the modified protein. To our knowledge, this is the first report where VSV-G was successfully engineered to include a large (253 amino acids) exogenous peptide and where attempts were made to change the infection profile of VSV-G pseudotyped vectors.

## Background

Retroviruses, including lentiviruses, integrate into the genome of host cells, and the expression of the transduced genes can persist throughout cell divisions. Hence, murine leukemia virus (MLV)- and lentivirus-based vectors are among the most commonly used tools for gene transfer in eukaryotic cells in the laboratory, and may one day become clinically important. Lentiviral vectors have also the additional advantage of transducing non-dividing cells, which broadens their application to both resting and terminally differentiated cells.

Despite continuous improvement of retroviral and lentiviral gene transfer over the past years [1-3], the current inability to target infection to cells of interest remains a severe limitation, preventing the development of efficient, safe and cost-effective clinical application. A number of reports have already been published to this end (for review, see [4-6]). The majority of these studies were attempts to redirect the tropism of the ecotropic envelope glycoprotein (GP) of MLVs by the addition of ligand motifs, which bind to specific molecules associated with the cell membrane. However, these approaches generally met with limited success. Although the engineered viruses usually did bind to the new receptors, infection titres were low. Inefficient transduction was mostly due to diminished fusion activity of the engineered GP, which consequently prevented infectious translocation of the viral capsids into cells [7-9].

Retroviral and lentiviral GPs are made of two parts, produced from the same precursor following proteolytic maturation. SU, or surface protein, recognises the viral receptor, and TM, the transmembrane protein, carries the fusion activity and tethers the GP to virions [4-6]. However, retroviruses and lentiviruses can be pseudotyped by a number of GPs from other viruses, such as the hemagglutinin (HA) of influenza virus, the envelope proteins (E1 and E2) of Sindbis virus and the G protein of vesicular stomatitis virus (VSV-G). These have all higher fusion activity than the native GPs and remain tightly attached to virions. HA has already been engineered for targeting purposes through N-terminal addition of various ligands, of which one successfully redirected MLV tropism towards human melanoma cells [10]. E2 has also been genetically modified to display the immunoglobulin-binding domain of *Staphylococcus aureus *protein A [11]. After addition of antibodies specific for certain cell membrane markers, a relatively efficient retargeted infection of pseudotyped MLV- and HIV based vectors was observed *in vitro *[11], as well as *in vivo *[12]. Recently, E2 was engineered to include a scFv against CCR5, which specifically directed lentiviral vectors to CCR5-expressing cells [13].

These findings are promising for future vector modifications, although HA and the Sindbis proteins are seldom used for gene transfer protocols. Due to its broad tropism and stability, VSV-G, on the other hand, is the most widely used protein for pseudotyping retroviral and lentiviral vectors [14,15]. VSV-G is a trimerised transmembrane molecule, although its exact structure is not fully known. Moreover, its ligand has not been identified [16], which hampers rational design of targeting strategies. Additionally, only a few permissible sites for short (2–10 amino acids) peptide insertions have been isolated [17-20]. Nevertheless, these studies all confirmed that VSV-G might be amenable to genetic engineering for targeting purposes. Guibinga *et al *inserted a 10 amino acid collagen-binding peptide close to the N-terminal of VSV-G, and could show specific attachment of MLV- and HIV-1-based vectors to collagen matrix [17]. To date, however, no redirected cell transduction has been reported. We therefore attempted to target infection by attaching a large ligand binding domain, an scFv against MHC-I, directly in the N-terminal of the protein, a site that Yu and Schaffer confirmed permissive. We show that the novel GP, with its large exogenous peptide, (i) is processed and transported to the cell surface, (ii) provides a new binding specificity but (iv) transduces target cells very inefficiently, although better than control scFv/VSV-G. We speculate that this is due to an inefficient fusion activity, and discuss potential improvements.

## Results and discussion

**Figure 1:**
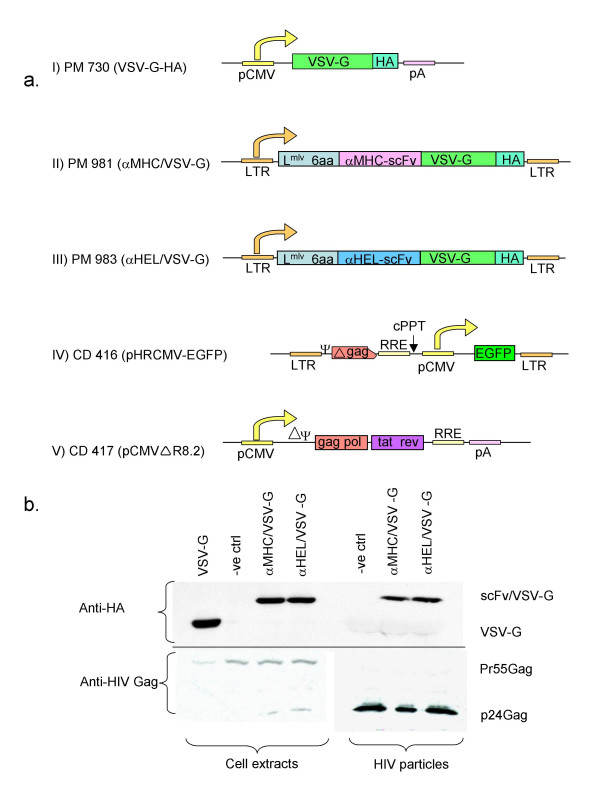
scFv-VSV-G expression plasmids and incorporation in HIV-1 derived particles. a) Expression plasmids: (I) VSV-G expression plasmid (PM 730). The 1.6 kB HindIII-BamHI VSV-G fragment (serotype Indiana) was transferred from pFB.VSVG (J.M. Heard, Paris, France) into pcDNA3 (InVitrogen) by PCR cloning according to standard procedures. A haemagglutinin (HA) sequence was added at the C terminus of VSV-G for immunodetection. (II and III) scFv/VSV-G expression plasmids. The chimaeric constructs were generated by PCR-based cloning. Mature VSV-G (from amino acid 17) was amplified from PM 730 and introduced into the PM 441 and PM 442 plasmids [23]. These constructs originate from an MLV-derived plasmid (FBMOSALF [31]), modified to contain a scFv (αMHC and αHEL, respectively [27]), upstream of the GP gene. Consequently, the resulting constructs (αMHC/VSV-G and αHEL/VSV-G) express the genes from the MLV LTR, with a MLV leader sequence (L^mlv^) and 6 additional amino acids from the virus. (IV and V) Vectors for production of HIV-1 derived viral particles. A HIV-1-based lentiviral vector (V) (CD 416; pHRCMV) [29], into which EGFP gene had been inserted, together with a helper plasmid (CD 417; pCMV△R8.2) [29] expressing Gag, Pol and accessory HIV-1 proteins (IV), were used for production of HIV-1 particles. Expression vectors, physical maps and primer sequences are available upon request. b. VSV-G- and scFv/VSV-G pseudotyped HIV-1 particles, produced in 293T cells. 2 × 10^6 ^293T cells in 10 cm diameter tissue culture dishes were transiently transfected with 5 μg of an LTR-driven EGFP vector (pHRCMV-EGFP) and 4 μg of a helper plasmid (pCMV△R8.2) [29], using the calcium phosphate co-precipitation procedure [36]. 5 μg PM 730 or 30 μg scFv/VSV-G plasmid (PM 981 or PM 983) were also included. DNA precipitates were removed after 16 hours, and the viral supernatants were collected 24–48 hours later and pelleted by ultracentrifugation (BeckmanCoulter) at 25 kRPM, 4°C for 2 hours and resuspended in 1% of the original volume. Cell lysates and 2 μl concentrated scFv/VSV-G virus or 10 μl of non-concentrated VSV-G virus were separated on a 12 % SDS polyacrylamide gel and transferred onto Protran nitrocellulose membranes (Schleicher and Schuell). HA-tagged VSV-G and scFv/VSV-G were detected using a rat anti-HA antibody (Sigma), followed by horse radish peroxidase (HPO) conjugated anti-RatIgG (Dako). p24Gag was detected using SF2 rabbit monoclonal antibody (NIH AIDS Research and Reference Reagent Program) followed by anti-rabbit IgG/HPO (Santa Cruz), and was used as an internal reference to normalise for the virion protein quantities. The membranes were developed with Renaissance chemoluminescence kit (NEN Life Science Products), as recommended by the supplier.

As a model system, we decided to target MHC-I molecules on human cells, as these membrane receptors can mediate cell infection by retroviral and lentiviral vectors [11,21-23]. As already described [23], a scFv against MHC-I (αMHC) consists of the heavy and light chain variable regions of a mouse monoclonal antibody (B9.12.1) [24], coupled by flexible spacer. This peptide was fused to the N-terminal of the mature coding sequence of VSV-G (αMHC/VSV-G). Although certain anti-MHC-I monoclonal antibodies are known to inhibit HIV production, B9.12.1 appears to have a minor effect on the viral life cycle [25]. As a control, we used a similar construct, containing an anti-hen egg lysozyme scFv (αHEL) [26], which does not recognise any surface markers on human cells. For immunodetection purposes, the C-terminal of the VSV-G cDNA was fused to an HA sequence. The two chimaeras were obtained by inserting the HA-containing VSV-G cDNA downstream of scFv sequences in vectors originating from Moloney MLV constructs [27]. Consequently, the leader sequence from Moloney MLV GP is used, and 6 aminoacids from the original GP are retained between the scFv and VSV-G (Figure 1).

VSV-G is glycosylated, folded and trimerised in the endoplasmatic reticulum prior to export to the Golgi [28]. Changes in the protein structure often results in inappropriate processing [18] (our own unpublished observations). We therefore assessed the intracellular distribution of the scFv/VSV-G molecules in transfected HeLa cells, revealed by a rat anti-HA antibody. HA-tagged VSV-G and scFv/VSV-G proteins were all found scattered throughout the cells and a fraction of the protein were detected in, or very close to the cellular membrane (data not shown). With the conformation-specific anti-VSV-G antibody 8G5F11 (a generous gift by Dr D. Lyles), VSV-G and scFv/VSV-G molecules were also detected by flow cytometry on the surface of transfected HeLa and 293T cells, implying that the engineered VSV-G proteins retain conformational resemblance to the native molecule. Therefore, we have succeeded in generating correctly processed hybrid proteins, which is in accord with a recent report that showed that the N-terminal of VSV-G is permissive for short peptide insertion [20]. We next assessed the incorporation of the chimaeras into lentiviral particles. To this aim, expression vectors for VSV-G, αMHC/VSV-G and αHEL/VSV-G were co-transfected with the pCMV△R8.2 helper plasmid expressing Gag, Pol and accessory HIV-1 proteins together with pHRCMV-EGFP HIV-1-based lentiviral vector [29]. Viral particles were prepared from culture supernatants and analysed by immunoblotting for the presence of VSV-G proteins. As shown in Figure 1B, the αMHC/VSV-G and αHEL/VSV-G chimaeras were incorporated in HIV-1 recombinant particles at levels reflecting those in the transfected cells. There was, however, slightly lower amounts of the chimaeric proteins versus the parental version in transfected cells, which may be a result of decreased synthesis (different expression plasmids) or reduced stability of the new molecules.

**Figure 2:**
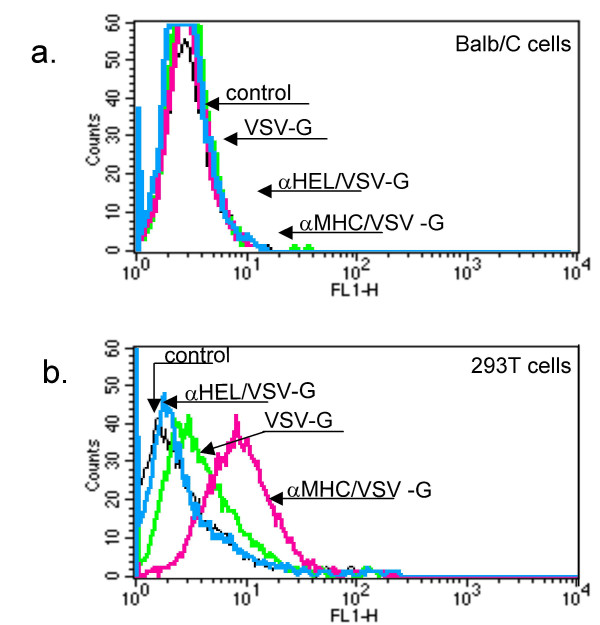
Binding of VSV-G- or scFv/VSV pseudotyped HIV-1 particles to target cells. 5 × 10^5 ^293T or Balb/C cells were incubated with 1 ml (non-concentrated) pseudotyped HIV-1 particles from transiently transfected 293T cells for 30 minutes on ice. Cells were washed twice with phosphate buffered saline (PBS, pH 7.0) and incubated in block buffer (BB: 10% bovines serum albumine, 0.1 M Glycine in PBS (pH 7.0)) for 30 minutes on ice, which was then replaced by 200 μl of 5G8F11 hybridoma supernatant, kindly donated by Dr Douglas Lyles (Winston-Salem NC, US). After 1 hour on ice, the cells were washed twice with BB and resuspended in 100 μl fluorescein isothiocyanate-conjugated anti-mouse IgG antibody (FITC-Ab) (Sigma), diluted 100 times in BB. The cells were rinsed again after 1 hour, fixed with 0.2 % formaldehyde and analysed using a FACScalibur fluorescence-activated cell sorter (Becton Dickinson).

Next, we investigated whether αMHC/VSV-G could mediate specific viral binding to human cells. HIV-1-derived particles were pseudotyped with either VSV-G or the scFv/VSV-G molecules and placed in the presence of murine Balb/C fibroblasts or of human 293T cells, which were then analysed by flow cytometry. No viral binding to mouse cells was seen with any of the pseudotyped vectors (Figure 2). It is possible that the scFvs had masked/inactivated the natural receptor-binding site of VSV-G. However, the lack of VSV-G binding is puzzling, as the protein efficiently drives infection of most cell types. Although not quantified precisely, the affinity of VSV-G for its receptor is presumably low, as maximal binding of radiolabelled VSV to Vero cells was shown to require 12 hours of incubation at 4°C [30]. Hence, we suggest that VSV-G pseudotyped viral particles bind to cells by low-affinity attachment which does not resist thorough washing steps. As for human cells, VSV-G and αHEL/VSV-G gave both poor binding signals, reminiscent of what was observed with mouse fibroblasts, whereas αMHC/VSV-G bound well to target cells. This is in agreement with the binding of natural HA, which attached to cells less strongly than its ligand-modified variants [10]. Taken together, our data suggests that αMHC/VSV-G can mediate specific and robust attachment to human cells via MHC class I. However, it does not exclude that scFV/VSV-G chimaeras cannot bind to the VSV-G receptor, as binding of native VSV-G could not be visualised.

Having found the selective binding properties of αMHC/VSV-G molecules, we assessed if the virus would discriminatingly infect human cells. αMHC/VSV-G and αHEL/VSV-G pseudotyped EGFP expressing HIV-1 derived particles were used to infect human or mouse cells. However, we observed a dramatic drop in infectivity with the modified VSV-G molecules as compared to the native VSV-G. To distinguish between reduced fusion activity and binding is difficult, as we were not been able to quantify the binding of VSV-G to cells. However, αMHC/VSV-G attaches to human cells, but the fusogenicity is very poor, as shown by analysis of syncytium formation in transfected HeLa cells (data not shown). This is suggestive of partly dysfunctional fusion machinery, although some activity remains, as these proteins still mediate infection significantly better than bald viral particles. To properly titre the αMHC/VSV-G and αHEL/VSV-G pseudotyped viruses, the particles were concentrated by ultracentrifugation (x100). αHEL/VSV-G pseudotyped particles retain some infective activity (Table 1), as these vector preparations are still more infective (x10) than bald (VSV-G negative) viruses. αMHC/VSV-G pseudotyped HIV-1 transduces 293T cells more efficiently than αHEL/VSV-G, but is significantly lower than VSV-G. However, the two VSV-G chimaeras were similarly unsuccessful in infecting murine cells. Although inefficient, we have engineered a molecule that satisfies the selective criteria as it can mediate preferential infection of a certain cell type.

**Table 1:** Infection assay on human cells.

Virus	Exp 1	Exp 2	Exp 3	Exp 4	Exp 5	Exp 6	Exp 7
αMHC/VSV-G	7200	3020	3620	2880	1400	1720	1800
αHEL/VSV-G	1120	620	760	380	260	360	420

Supernatant from HIV-1-producing 293T cells were passed through a 0.45 μm filter (Sarstedt). αMHC/VSV-G and αHEL/VSV-G HIV-1 samples were concentrated 100 times by centrifugation (25 kRPM at 4°C for 2 hours in a BeckmanCoulter ultracentrifuge) and were resuspended in 1% bovine serum albumine. VSV-G pseudotyped HIV-1-derived viral particles were directly used without prior concentration to infect target cells. When required, the virus was stored at -80°C. 50% confluent target cells, either human 293T (depicted) and HeLa cells, mouse *Mus Dunni *cells or monkey Cos-7 cells (not shown), were cultured with dilutions of virus for 16 hours in the presence of 5 μg/ml polybrene. 48 hours later, green fluorescent colonies were counted. Titres (colony forming units (cfu)/ml) on infected 293T cells of αMHC/VSV-G and αHEL/VSV-G particles from 7 independent experiments are shown. VSV-G pseudotyped HIV-1 was used as control for successful virus production and infection, and generally gave titers of 10^7^-10^6 ^cfu/ml (data not shown).

**Figure 3:**
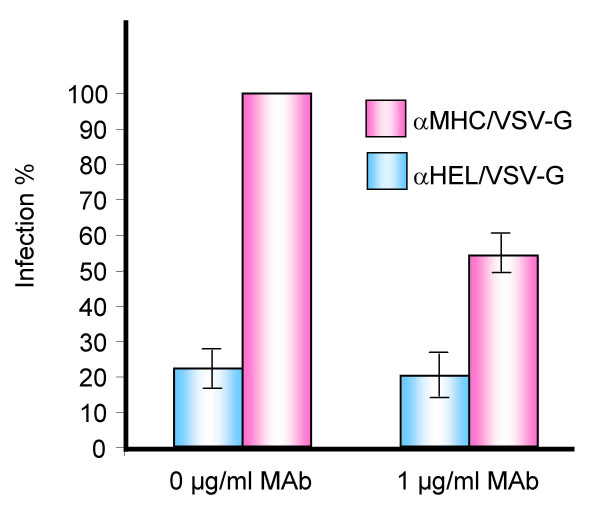
Inhibition of infection with an anti-MHC antibody. Supernatant from HIV-1-producing 293T cells were passed through a 0.45 μm filter (Sarstedt). αMHC/VSV-G and αHEL/VSV-G HIV-1 samples were concentrated 100 times by centrifugation (25 kRPM at 4°C for 2 hours in a BeckmanCoulter ultracentrifuge) and were resuspended in 1% bovine serum albumine. 70% confluent target cells (293T cells) were treated with 1.0 μg/ml B9.12.2 mAb for 30 minutes prior to infection with concentrated αMHC/VSV-G or αHEL/VSV-G pseudotyped virions for 16 hours in the presence of 5 μg/ml polybrene. 48 hours later, EGFP positive clones (colony forming units (cfu)/ml) were counted. 100% transduction corresponds to the cfu obtained by the αMHC/VSV-G particles after pre-treatment of an isotype-matched antibody control. The results are representative of four independent experiments, and error bars indicate the standard error of the mean.

To confirm that the selective infection of human cells by the αMHC/VSV-G virus was attributed to the targeting scFv, we blocked the MHC-1 molecules on the human cells with the monoclonal antibody B9.12.1 prior to infection. A 50% loss in infectivity by the αMHC/VSV-G HIV-1 particles was observed with the highest concentration of MAb (1 μg/ml) (Figure 3). The αHEL/VSV-G control virus, already with a very low titre, was not affected by the presence of the antibody (Figure 3). Also, the infectivity of the VSV-G pseudotyped virions remained unchanged when the target cells were pre-treated with the antibody (data not shown). That we did not succeed in preventing all infection events with an excess of antibody is difficult to explain. It is possible that the natural turnover of MHC-I allows recycled molecules to appear on the surface, available for viral binding. Similarly, Marin *et al *could not completely inhibit the infection of MHC-I targeted MLV virus with the same antibody [23]. Although not complete, we show that by blocking the targeted molecule, the titre of αMHC/VSV-G virus can be reduced, suggesting that the infectivity is dependent on the exogenous cell directing peptide.

This is the first demonstration of a directed, albeit still inefficient, VSV-G based transduction system. Improvement of titres may be achieved by including flexible [13] or cleavable linkers between the subunits in the chimaeric molecule. Also, replacing the αMHCI scFv with other cell targeting peptides will be important to validate the potency of this targeting model. If successfully improved, this prototype may bear fruit in future gene therapy studies.

## Conclusion

To selectively deliver transgenes into target cells could be of interest when utilising gene transfer vectors. GPs of different viruses have been modified to meet this end. However, VSV-G, the most commonly used pseudotyping protein for retro- and lentiviral vectors, has not yet been successfully adapted for directed gene delivery. Recently, reports have shown that this protein is indeed amenable to small peptide insertions. Here, we expend this by linking a large (253 aa) cell-directing scFv directly to its N-terminal. These hybrid proteins are processed and get transported to the surfaces of transfected cells. They are also capable of pseudotyping lentiviral particles, which are shown to specifically attach to target cells. However, the fusogenicity of the novel proteins are diminished and the resulting titres of the viral particles are reduced. Nevertheless, on specific target cells, the infectivity is still higher than with the control vector. This is the first demonstration of a directed, albeit still inefficient, VSV-G based transduction system. Importantly, we show that VSV-G can accept large peptic additions in its N-terminal, which should encourage further improvements. Hence, this prototype may bear fruit in future gene therapy studies.

## Materials and methods

### Engineering of VSV-G and scFv/VSV-G expression plasmids

The 1.6 kB HindIII-BamHI VSV-G fragment (serotype Indiana) was transferred from pFB. VSV-G into pcDNA3 (InVitrogen). To introduce a HA tag in the C terminal of VSV-G, the cDNA was amplified with a T7-specific sense primer (5' TAATACGATCACTTTAGGG) and an antisense oligo, including the HA sequence (in miniscule), a stopcodon and an *Xho site *(5' CCC*CTCGAG*TTA agcgtaatcaggaacatcataaggata CTTTCCAAGTCGGTTCATCTC). The product was digested with HindIII and XhoI, and reinserted into pcDNA3.

To generate scFv/VSV-G molecules, the sequence for mature VSV-G (from amino acid 17) was amplified with a sense primer, also containing a *NotI *site and an additional nucleotide to retain the reading frame (5' CCC*GCGGCCGC*AAAGTTCACCATAGTTTTTCCACAC). The anti-sense primer hybridises to the HA sequence, contains a stopcodon and carries a *Cla I *site (5' CCC*ATCGAT *TTAAGCGTAATCAGGAACATCATA). The NotI/ClaI restricted PCR product was ligated into NotI/ClaI-cleaved PM441 and PM442 plasmids [23]. These constructs originate from an MLV-derived plasmid (FBMOSALF [31]), modified to contain an scFv (αMHC and αHEL, [27]), upstream of the GP gene. Consequently, the resulting constructs (αMHC/VSV-G and αHEL/VSV-G) express the gene from the MLV LTR, with a MLV leader sequence and 6 additional amino acids from the virus (see Fig 1).

Restriction enzymes were purchase from Roche or Invitrogene and all oligonucleotides were obtained from Sigma.

### Cells and culture conditions

HeLa [32], 293T [33], TelCeb6 [31], Cos-7 [34] and *Mus Dunni *cells [35] were grown at 37°C in Dulbecco's modified Eagle's medium (Sigma), supplemented with 10% heat inactivated foetal calf serum (Gibco), 100 units/ml streptomycin, 100 units/ml penicillin and 2 mM L-glutamine in a humified 5% CO_2 _incubator.

### Transient expression of αMHC/VSV-G and αHEL/VSV-G

HeLa or 293T cells were seeded on 6-well plate at 60% confluency. The following day, cells were transiently transfected using the classic CaPO_4 _co-precipitation method [36] with 5 μg DNA/well. The precipitate was removed and gene expression was confirmed 24–48 hours later by Western Blot, immunofluorescence or flow cytometry.

### Production of VSV-G and scFv/VSV-G pseudotyped lentiviral particles

To express HIV-1 particles, 293T cells (75% density) in a 10-cm culture plate were transiently transfected with 5 μg of an LTR-driven EGFP vector (pHRCMV-EGFP) and 4 μg of a helper plasmid (pCMV△8.2) [29]. 5 μg VSV-G or 30 μg scFv/VSV-G plasmids were also included. DNA precipitate was removed after 16 hours, and the viral supernatants were collected 24–48 hours later.

### Immunoblotting assays of VSV-G and scFv/VSV-G

For virion protein preparation, 1 ml of culture supernatant from virus producing cells were adjusted to 10 mM CaCl_2 _and left at room temperature for 30 minutes. Precipitated viruses were spun down at 13 k rpm at 4°C for 1 minute and resuspended in 50 μl of electrophoresis loading buffer. Cells were resuspended in triplex lysis buffer (50 mM Tris-HCl pH8.0, 150 mM NaCl, 0.2% NaN_3_, 0.1% SDS, 1% NP40, 0.5% Na-deoxycholate, 2 mg/ml leupeptin, 1 mM phenylmethyl sulfone fluoride) and left on ice for 30 minutes. Cell debris and nuclei were removed by centrifugation (13 k rpm at 4°C for 10 minutes). The samples were fractionated through SDS polyacrylamide (10%) gels (SDS-PAGE) and transferred to Protran nitrocellulose membranes (Schleicher and Schuell). VSV-G and scFv/VSV-G carry an HA tag, and were detected by a rat anti-HA antibody (Sigma), followed by a horseradish peroxidase (HPO) conjugated anti-RatIgG (Dako). p24Gag, detected by SF2 rabbit monoclonal antibody (NIH AIDS Research and Reference Reagent Program) and an anti-rabbit IgG/HPO (Santa Cruz), was used as an internal reference to normalise the virion proteins. The membranes were developed with Renaissance chemoluminescence kit (NEN Life Science Products), as recommended by the supplier.

### Detection of scFv/VSV-G by immunofluorescence assay

Transfected HeLa or 293T cells were incubated with a conformation specific anti-VSV-G antibody (5G8F11, a generous gift by Dr Douglas Lyles, Winston-Salem) for 30 minutes, washed and revealed by a fluorescein isothiocyanate conjugated anti-mouse IgG antibody (FITC-anti-MuIg; Sigma). VSV-G expressing cells were detected under a fluorescence microscope (Zeiss).

Distribution of intracellular, HA-tagged VSV-G was assessed in paraformaldehyde-fixed, Triton-X permeabilised transfected HeLa cells, grown on cover slips. The proteins were visualised with a rat anti-HA antibody together with a FITC labelled anti-Rat IgG (both Sigma), and analysed with a confocal microscope (Leica).

### Detection of scFv/VSV-G by flow cytometry

2 × 10^5 ^transfected 293T cells were collected in phosphate buffered saline (PBS), incubated in block buffer (BB: 10% bovines serum albumin, 0.1 M Glycine in PBS) for 30 minutes on ice, which was replaced by 200 μl of 5G8F11 hybridoma supernatant. After 1 hour on ice, the cells were washed twice with BB and resuspended in 100 μl FITC-anti-MuIg. The cells were rinsed again after 1 hour, fixed with 0.2 % formaldehyde and analysed on a FACScalibur fluorescence-activated cell sorter (Becton Dickinson).

### VSV-G binding assays

5 × 10^5 ^human 293T and HeLa cells, and *Mus Dunni *cells were incubated with 1 ml pseudotyped HIV-1 particles from transiently transfected 293T cells for 30 minutes on ice. Cells were washed two times with PBS. scFv/VSV-Gs or VSV-G, attached to the cell surfaces, were detected as previously described.

### Infection assays

Supernatant from HIV-1-producing 293T cells were passed through a 0.45 μm filter (Sarstedt). Some samples were concentrated 100 times by centrifugation (25 k rpm at 4°C for 2 hours in a BeckmanCoulter ultracentrifuge) and were carefully resuspended in 1% BSA. When required, the virus was stored at -80°C.

50% confluent target cells, either human 293T and HeLa cells, mouse *Mus Dunni *cells or monkey Cos-7 cells, were cultured with dilutions of virus for 16 hours in the presence of 5 mg/ml polybrene. 48 hours later, green fluorescent colonies were counted or cells were analysed by flow cytometry.

To block αMHC/VSV-G driven infection, target cells were preincubated with the B9.12.1 (< 1 μg/ml, Beckman Coulters) for 30 minutes before addition of the virus.

## Competing interests

The author(s) declare that they have no competing interests.

## Authors' contributions

HD participated in the design of the project, carried out the practical work and drafted the manuscript. MP conceived and managed the project.

## References

[B1] Lundstrom K (2003). Latest development in viral vectors for gene therapy. Trends Biotechnol.

[B2] Gould DJ, Favorov P (2003). Vectors for the treatment of autoimmune disease. Gene Ther.

[B3] Sinn PL, Sauter SL, McCray PBJ (2005). Gene therapy progress and prospects: development of improved lentiviral and retroviral vectors--design, biosafety, and production. Gene Ther.

[B4] Sandrin V, Russell SJ, Cosset FL (2003). Targeting retroviral and lentiviral vectors.. Curr Top Microbiol Immunol.

[B5] Lavillette D, Russell SJ, Cosset FL (2001). Retargeting gene delivery using surface-engineered retroviral vector particles.. Curr Opin Biotechnol.

[B6] Karavanas G, Marin M, Salmons B, Gunzburg WH, Piechaczyk M (1998). Cell targeting by murine retroviral vectors. Crit Rev Oncol Hematol.

[B7] Zhao Y, Zhu L, Lee S, Li L, Chang E, Soong NW, Douer D, Anderson WF (1999). Identification of the block in targeted retroviral-mediated gene transfer.. Proc Natl Acad Sci U S A.

[B8] Benedict CA, Tun RY, Rubinstein DB, Guillaume T, Cannon PM, Anderson WF (1999). Targeting retroviral vectors to CD34-expressing cells: binding to CD34 does not catalyze virus-cell fusion. Hum Gene Ther.

[B9] Karavanas G, Marin M, Bachrach E, Papavassiliou AG, Piechaczyk M (2002). The insertion of an anti-MHC I ScFv into the N-terminus of an ecotropic MLV glycoprotein does not alter its fusiogenic potential on murine cells. Virus Res.

[B10] Hatziioannou T, Delahaye E, Martin F, Russell SJ, Cosset FL (1999). Retroviral display of functional binding domains fused to the amino terminus of influenza hemagglutinin. Hum Gene Ther.

[B11] Morizono K, Bristol G, Xie YM, Kung SK, Chen IS (2001). Antibody-directed targeting of retroviral vectors via cell surface antigens.. J Virol.

[B12] Morizono K, Xie Y, Ringpis GE, Johnson M, Nassanian H, Lee B, Wu L, Chen IS (2005). Lentiviral vector retargeting to P-glycoprotein on metastatic melanoma through intravenous injection. Nat Med.

[B13] Aires da Silva F, Costa MJ, Corte-Real S, Goncalves J (2005). Cell type-specific targeting with sindbis pseudotyped lentiviral vectors displaying anti-CCR5 single-chain antibodies. Hum Gene Ther.

[B14] Lu X, Humeau L, Slepushkin V, Binder G, Yu Q, Slepushkina T, Chen Z, Merling R, Davis B, Chang YN, Dropulic B (2004). Safe two-plasmid production for the first clinical lentivirus vector that achieves >99% transduction in primary cells using a one-step protocol. J Gene Med.

[B15] Quinonez R, Sutton RE (2002). Lentiviral vectors for gene delivery into cells. DNA Cell Biol.

[B16] Coil DA, Miller AD (2004). Phosphatidylserine is not the cell surface receptor for vesicular stomatitis virus. J Virol.

[B17] Guibinga GH, Hall FL, Gordon EM, Ruoslahti E, Friedmann T (2004). Ligand-modified vesicular stomatitis virus glycoprotein displays a temperature-sensitive intracellular trafficking and virus assembly phenotype.. Mol Ther.

[B18] Li Y, Drone C, Sat E, Ghosh HP (1993). Mutational analysis of the vesicular stomatitis virus glycoprotein G for membrane fusion domains. J Virol.

[B19] Schlehuber LD, Rose JK (2004). Prediction and identification of a permissive epitope insertion site in the vesicular stomatitis virus glycoprotein.. J Virol.

[B20] Yu JH, Schaffer DV (2006). Selection of novel vesicular stomatitis virus glycoprotein variants from a peptide insertion library for enhanced purification of retroviral and lentiviral vectors. J Virol.

[B21] Roux P, Jeanteur P, Piechaczyk M (1989). A versatile and potentially general approach to the targeting of specific cell types by retroviruses: application to the infection of human cells by means of major histocompatibility complex class I and class II antigens by mouse ecotropic murine leukemia virus-derived viruses. Proc Natl Acad Sci U S A.

[B22] Etienne-Julan M, Roux P, Carillo S, Jeanteur P, Piechaczyk M (1992). The efficiency of cell targeting by recombinant retroviruses depends on the nature of the receptor and the composition of the artificial cell-virus linker. J Gen Virol.

[B23] Marin M, Noel D, Valsesia-Wittman S, Brockly F, Etienne-Julan M, Russell S, Cosset FL, Piechaczyk M (1996). Targeted infection of human cells via major histocompatibility complex class I molecules by Moloney murine leukemia virus-derived viruses displaying single-chain antibody fragment-envelope fusion proteins. J Virol.

[B24] Rebai N, Malissen B (1983). Structural and genetic analyses of HLA class I molecules using monoclonal xenoantibodies. Tissue Antigens.

[B25] Briant L, Benkirane M, Girard M, Hirn M, Iosef C, Devaux C (1996). Inhibition of human immunodeficiency virus type 1 production in infected peripheral blood mononuclear cells by human leukocyte antigen class I-specific antibodies: evidence for a novel antiviral mechanism.. J Virol.

[B26] Ward ES, Gussow D, Griffiths AD, Jones PT, Winter G (1989). Binding activities of a repertoire of single immunoglobulin variable domains secreted from Escherichia coli. Nature.

[B27] Russell SJ, Hawkins RE, Winter G (1993). Retroviral vectors displaying functional antibody fragments.. Nucleic Acids Res.

[B28] Coll JM (1995). The glycoprotein G of rhabdoviruses. Arch Virol.

[B29] Naldini L, Blomer U, Gallay P, Ory D, Mulligan R, Gage FH, Verma IM, D. T (1996). In vivo gene delivery and stable transduction of nondividing cells by a lentiviral vector.. Science.

[B30] Schlegel R, Sue TT, Willingham MC, Pastan I (1983). Inhibition of VSV binding and infectivity by phosphatidylserine: is phosphatidylserine a VSV-bidning site?. Cell.

[B31] Cosset FL, Takeuchi Y, Battini JL, Weiss RA, Collins MK (1995). High-titer packaging cells producing recombinant retroviruses resistant to human serum. J Virol.

[B32] Scherer WF, Syverton JT, Gey GO (1953). Studies on the propagation in vitro of poliomyelitis viruses. IV. Viral multiplication in a stable strain of human malignant epithelial cells (strain HeLa) derived from an epidermoid carcinoma of the cervix.. J Exp Med.

[B33] Graham FL, Smiley J, Russell WC, Nairn R (1977). Characteristics of a human cell line transformed by DNA from human adenovirus type 5.. J Gen Virol.

[B34] Gluzman Y (1981). SV40-transformed simian cells support the replication of early SV40 mutants.. Cell.

[B35] Lander MR, Chattopadhyay SK (1984). A Mus dunni cell line that lacks sequences closely related to endogenous murine leukemia viruses and can be infected by ectropic, amphotropic, xenotropic, and mink cell focus-forming viruses.. J Virol.

[B36] Sambrook J, Fritsch E, Maniatis T (1989). Molecular cloning: a laboratory manual. Cold Spring Harbor Laboratory Press.

